# S114. ASSOCIATION BETWEEN APATHY AND DEPRESSION: SECONDARY OR REFLECTING UNDERLYING COMMON FEATURES?

**DOI:** 10.1093/schbul/sby018.901

**Published:** 2018-04-01

**Authors:** Ann Faerden, Siv Hege Lyngstad, Ingrid Melle

**Affiliations:** Oslo University Hospital

## Abstract

**Background:**

Negative symptoms and depression have in many studies been found to be moderately associated, and when present, it reflects negative symptoms of a secondary nature. Primary negative symptoms are thought to be intrinsic to schizophrenia, while secondary to be caused by depression, positive symptoms and medication side effects. Instability in above associations over time is also considered to reflect a secondary origin. Despite the clinical importance of secondary negative symptoms little research has focused on what is their underlying nature. Especially apathy and depression have common clinical features and are often found to correlate. Apathy and depression are self-perceived states and self-reports could add to our understanding. Studying the underlying themes of associations between apathy and depression as well as stability over time could therefore add to the current understanding of the primary or secondary nature of negative symptoms.

**Methods:**

Eighty-four first episode psychosis patients from TOP/NORMENT study in Oslo, Norway were assessed at baseline and 1-year follow-up with the Calgary Depression Scale (CDSS), Apathy Evaluation Scale (AES), both self-report (AES-S) and clinician (AES-C), and the Positive and Negative Symptoms Scale (PANSS). Correlation with total scale, individual scale items and linear regression was used to study associations and explained variance over time. Results were repeated controlling for positive symptoms and excluding those with high level of depression.

**Results:**

CDSS and AES correlated at the 0.4 to 0.5 levels at both baseline and follow up, regardless of AES-S or AES-C. Hopelessness and feeling of depression were the CDSS items with stable and concurrent correlation strength to AES-S and AES-C. For CDSS, we found correlation of equal strength and stability to the AES-S- and AES-C items of getting things done during the day, spending time on interests, getting excited and taking initiative. Same significant correlations to CDSS were found for PANSS amotivation factor, but not for PANSS expressive factor. Controlling for PANSS positive symptoms did not change results, and excluding those with high levels of depression only mildly changed results.

**Discussion:**

This study shows a significant correlation between apathy and depression that is stable over time for the full scale and also at the item level, regardless of self-reporting or clinician assessed apathy. Underlying themes of the concurrent correlation reflect lack of initiative and hopelessness and are in line with the defeatist beliefs found to correlate with negative symptoms, mediate between motivation and reduced effort and have recently been a target for cognitive remediation therapy. This study does not give an answer to a primary or secondary origin of apathy, but the stability points more to an underlying common nature than one being the cause of the other.

